# Do Overweight Adolescents Adhere to Dietary Intervention Messages? Twelve-Month Detailed Dietary Outcomes from Curtin University’s Activity, Food and Attitudes Program

**DOI:** 10.3390/nu7064363

**Published:** 2015-06-02

**Authors:** Kyla L. Smith, Deborah A. Kerr, Erin K. Howie, Leon M. Straker

**Affiliations:** 1 School of Public Health, Curtin University, GPO Box U1987, Perth 6845, Australia; E-Mail: d.kerr@curtin.edu.au; 2 School of Physiotherapy and Exercise Science, Curtin University, GPO Box U1987, Perth 6845, Australia; E-Mails: erin.howie@curtin.edu.au (E.K.H.); L.Straker@curtin.edu.au (L.M.S.)

**Keywords:** overweight, obese, adolescent, dietary intervention, adherence, dietary intake, dietary assessment, dietary patterns

## Abstract

Dietary components of adolescent obesity interventions are rarely evaluated with comprehensive reporting of dietary change. The objective was to assess dietary change in overweight adolescents, including adherence to dietary intervention. The dietary intervention was part of a multi-component intervention (CAFAP) targeting the physical activity, sedentary and healthy eating behaviors of overweight adolescents (*n* = 69). CAFAP was a staggered entry, within-subject, waitlist controlled clinical trial with 12 months of follow up. Diet was assessed using three-day food records and a brief eating behavior questionnaire. Changes in dietary outcomes were assessed using linear mixed models, adjusted for underreporting. Food record data suggested reduced adherence to dietary intervention messages over time following the intervention, despite conflicting information from the brief eating behavior questionnaire. During the intervention, energy intake was stable but favorable nutrient changes occurred. During the 12 month maintenance period; self-reported eating behaviors improved, energy intake remained stable but dietary fat and saturated fat intake gradually returned to baseline levels. Discrepancies between outcomes from brief dietary assessment methods and three-day food records show differences between perceived and actual intake, highlighting the need for detailed dietary reporting. Further, adherence to dietary intervention principles reduces over time, indicating a need for better maintenance support.

## 1. Introduction

Current rates of overweight and obesity in adolescence are concerning given the associated negative medical, psychosocial [1] and economic [2] consequences. Available evidence supports interventions with a comprehensive multi-disciplinary approach including a dietary component [3] and a family-based design with a focus on food and activity behaviors and attitudes [4]. Despite recommendations suggesting a focus on lifestyle, most interventions are evaluated using only measures of weight change. Few details about the implementation and evaluation of dietary interventions have been documented [5], making it difficult to understand how weight change may be achieved. Thus, a need for timely and detailed evaluation of adolescent obesity programs has been identified [4,6,7].

Surprisingly, few adolescent intervention trials have collected and reported detailed changes in participant dietary behaviors and intake data [8]. Some studies have not reported any dietary data [9,10], have not accounted for possible underreporting [11,12] or have used dietary assessment methods that provide only limited information and are restricted in their ability to detect true dietary change [13,14]. Further, measures of adherence to dietary interventions appear to be poorly described. In adolescent studies reporting dietary outcomes, the proportion of participants adopting specific dietary targets of the intervention (*i.e*., adherence) were not reported [11,12,14,15,16]. This is of particular concern as low adherence to dietary recommendations is a primary reason for poor outcomes following intervention [17]. Without adherence measures, it remains unclear how the dietary interventions create change in multi-disciplinary interventions [18].

To date, changes in diet following intervention have shown modest results, and long-term follow-up has been lacking [4]. In multi-disciplinary interventions where dietary data was collected, there have been improvements reported in some self-reported eating behaviors [15], or dietary intakes including reduction in total energy intake [14,16], absolute fat intake [12,16] and sugar intake [19]. Even these dietary findings have been limited by follow-up of less than 12 months [12,16,19] and a lack of adherence measures [12,14,15,16,19]. This very restricted evidence base limits the ability for future studies to replicate or compare dietary changes to determine the effectiveness of dietary interventions in overweight adolescents.

Against this background, the aim of this study was to comprehensively assess dietary change in overweight and obese adolescents for 12 months following an intervention (Curtin University’s Activity, Food and Attitudes Program) to better understand dietary change in this group. The assessment included analyses of adolescent adherence to the dietary component of the intervention (including changes in the primary intervention behavioral targets), changes in selected eating behavior strategies, and a detailed analysis of dietary nutrient intake as reported in three day food records.

## 2. Experimental Section

### 2.1. Study Design

**Figure 1:**
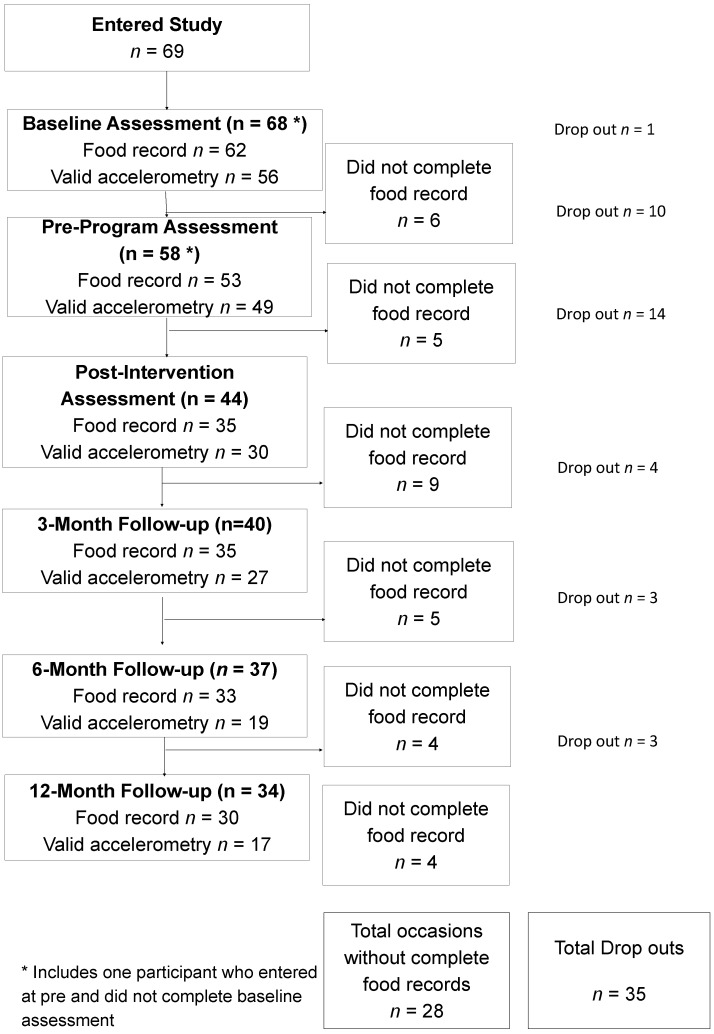
Participant numbers and food record completion during the waitlist controlled trial of Curtin University’s Activity, Food, and Attitudes Program.

This study was a multiple cohort, staggered-entry, waitlist period controlled clinical trial conducted at three sites in Western Australia (two metropolitan areas and one regional area) [20]. Briefly, overweight adolescents were recruited and assessed three months before the eight-week intensive phase of the intervention commenced, and assessed again immediately prior to the intervention. This method was chosen because it was considered unfair to withhold services from obese adolescents in view of the lack of appropriate treatment services available [21], and the dual pre-participation assessments allowed for a within-subjects control period. The staggered start for the seven cohort groups controlled for external seasonal and public event confounders to intervention effects. Further assessments were completed at the immediate conclusion of the eight-week program and again at three months, six months and 12 months post-intervention [20]. This trial was registered on the Australian New Zealand Clinical Trials Registry (ACTRN12611001187932). Figure 1 shows the progression of participants through the 17 months of the study. Each assessment time point is represented by a box on the left of the figure. In each box, the bold number refers to the number of adolescents potentially still available for each assessment, with the number of drop outs clearly stated on the right of the figure.

### 2.2. Participants

Between January 2012 and December 2013, 69 overweight or obese adolescents aged 11–16 participated in Curtin University’s Activity, Food and Attitudes Program (CAFAP). Participants were recruited via the health system, education system and from the general community and were screened by a medical practitioner for medical suitability prior to assessment. Further inclusion criteria was a BMI-for-age-and-sex above the 85th percentile [22]. Exclusion criteria included: obesity relating to an identified genetic, endocrine or metabolic disease, current treatment for psychiatric disorders or inability for parent and adolescent to attend twice weekly group sessions at a local community site. This study was approved by the Curtin University Human Ethics Research Committee (HR105/2011). Written informed assent/consent was obtained from all adolescents/parents.

### 2.3. Intervention

CAFAP was a community-based, multi-disciplinary healthy lifestyle program directed at overweight and obese adolescents and has been described in detail elsewhere [20,23]. The focus of CAFAP was increased physical activity, reduced sedentary behavior, reduced junk food intake and increased fruit and vegetable intake. The eight-week intensive phase of the intervention involved parents and adolescents and consisted of twice-weekly group sessions run by a psychologist, physiotherapist/exercise physiologist or dietitian. The intensive intervention period was followed by a tapered maintenance phase over 12 months.

### 2.4. Dietary Intervention

The dietary component of the intervention was facilitated by Accredited Practising Dietitians. Delivery style was guided by self-determination theory and goal setting theory, in line with the theoretical underpinnings of the intervention [23]. The dietary component focused on food groups rather than kilojoule intakes or specific nutrients. Participants learnt skills to help them make healthy food choices and were not provided with structured meal plans as recent evidence suggests that these are not well-received by adolescents [24]. The three primary nutrition intervention messages were: Eat more fruit; eat more vegetables; eat less junk food. The term ‘junk food’ is used to describe ‘discretionary’ foods that are considered energy-dense, nutrient-poor foods [25,26,27]. The dietary intervention consisted of 12 group education sessions with parents and adolescents together regarding general nutrition, energy balance, food labelling, diet variety, fast food, lunch box food, portion size and recipe modification, with the key messages reinforced in each session. Parents were also given practical training in buying healthy food during a supermarket visit and both parents and adolescents were involved in cooking classes focusing on the preparation of healthy foods containing fruits and vegetables. Tailored feedback on the adolescents’ diet, taken from the initial three day food record, was provided to each participant to assist with adolescent goal setting.

### 2.5. Dietary Assessment

#### 2.5.1. Nutrient Intake

Three day food records were used in this study to provide comprehensive descriptive information about meal patterns and intake of foods and beverages without extensive reliance on participant memory [28]. Records were completed at all six assessment points and used to assess changes in adolescent dietary intake. Three days provides a reasonable compromise between understanding the variation in daily adolescent diets [29] and the risk of poor quality information due to excessive participant burden [30,31]. Prior to completing the food record, adolescents were given training and written instructions from the research dietitian regarding estimating portion size and household measures. The adolescents were interviewed by the research dietitian to verify the completeness of the record and to probe for any forgotten food or beverages. Figure 1 shows the number of adolescents who actually completed food records at each time point. The food records were perceived by the adolescents as a burden to complete, and thus a small financial incentive was offered for detailed records. It is commonly accepted that adolescents lack motivation to complete food records and find them tedious to complete [30], so the relatively low numbers of non-completers is a positive outcome. 

Food records were analyzed using the NUTTAB 2010 and AUSNUT 2007 databases (FoodWorks Professional, Version 6, 2009; Xyris Software, Brisbane, Australia) for total energy, macronutrients and percentage contribution to energy intake, as well as intake of calcium and fiber. A food group analysis was also undertaken. Serving sizes for fruits and vegetables were derived from the Australian Guide to Healthy Eating, which specify that one serve of fruit is equivalent to 150 g and one serve of vegetables is equivalent to 75 g [27]. Servings of junk food (energy-dense, nutrient poor food) were equivalent to approximately 600 kilojoules [27]. For each participant, an average serve per day was calculated for fruits, vegetables and total junk food. The research dietitian completed all training, interviews and analysis of the food records. 

#### 2.5.2. Adherence to Intervention Messages

Adherence to the dietary intervention was measured by the percentage of participants who increased their intake of fruit and vegetables by at least 0.25 serves per day and reduced their intake of junk food by at least 0.5 serves per day, in line with the key dietary intervention messages. This was measured immediately post-intervention and 12 months post-intervention, using the data from three day food records. There is no accepted definition of a clinically important change in servings of key food groups, so this magnitude of change was chosen to reflect at least a 10% change in servings. This reflects the expected changes in physical activity and dietary behaviors following intervention as described in the protocol paper [20].

#### 2.5.3. Eating Behaviors

A short food behavior questionnaire based on validated questionnaires used in similar cohorts [32,33] was used to assess eating behaviors likely to be related to obesity. Questions included frequency of breakfast consumption, frequency of fast food consumption, frequency of eating meals as a family and sugar sweetened beverage consumption. Participants responded to questions about eating behavior frequency using a 5 point scale: Every day, 5–6 days per week, 3–4 days per week, 1–2 days per week, rarely or never. Questions regarding perceived intake of fruit, vegetables and junk food asked for the usual number of serves consumed each day, based on standard Australian serving size descriptions [27].

### 2.6. Statistical Analysis

Data were visually inspected for potential outliers and checks completed for individual data entry errors or implausible values. Tests for normality were conducted using histograms. Descriptive statistics at each assessment point are presented as mean ± standard deviations (SD). *t*-Tests were used to compare participants who completed the program with those who dropped out. All participants who participated in at least two occasions of data collection were included in the analysis. Adherence data is presented with additional separate results for those who completed all six occasions of data collection.

There is a high likelihood of underreporting by overweight and obese adolescents with food records [34,35]. In this study, implausible food records were identified using the ratio of energy intake (EI) to total energy expenditure (TEE) as a time-varying covariate [36] in the mixed model described below. Total energy expenditure was estimated using resting energy expenditure (REE) estimation equations [37] and activity energy expenditure (AEE) based on objectively measured accelerometry [38]. Where accelerometer data was unavailable (62 of 248 occasions) TEE was estimated as 0.0149 kcal/kg/min, based on the estimation equation validated by Puyau, Adolph, Vohra, Zakeri and Butte [38]. Underreporting (EI:TEE) was used as a time-varying covariate in the analysis of the self-reported questionnaire data and the dietary intake data from the food records.

Change in eating behaviors and dietary intake analysis: Linear mixed models were used to assess within-person changes in nutrient and eating behavior outcomes at the time points following conclusion of the eight-week intervention. Models included random intercepts to account for the within-person repeated measures. Slight deviations from normality were accounted for using bootstrapped resampling to estimate standard errors with 1000 replications. Underreporting ratios were included (EI:TEE) as time-varying covariates. To account for differences in the time between assessments, the monthly rate of change during each period was compared. The rate of change was calculated for the waitlist period (baseline to pre-intervention) and compared to the rate of change in outcome variables for all assessment periods between pre-intervention and 12 months post-intervention to assess intervention effectiveness. The analysis was completed using Stata/IC 13.0 for Windows (StataCorp LP, College Station TX, USA) and results were considered statistically significant at *p* < 0.05. No adjustment was made for multiple comparisons but 95% confidence intervals and *p*-values to three decimals places are reported.

## 3. Results

Based on the number of adolescents participating at each assessment point, a total of 281 diet records were possible. However, only 248 (88.3%) diet records were completed over the 17 months of data collection and were thus available for analysis. Following the intervention, participants increased their intake of fruit and reduced their intake of junk food as measured by three day food records, but vegetable intake did not change significantly [39]. As shown in Figure 1, 25 participants dropped out of the study between baseline and post-intervention and a further eight participants did not complete food records. This is similar to the relatively high dropout rates typically reported for healthy lifestyle programs aimed at overweight young people [40]. There were no differences at baseline between completers and non-completers, as discussed in the associated primary outcomes paper [39].

### 3.1. Adherence to Intervention Messages

**Figure 2:**
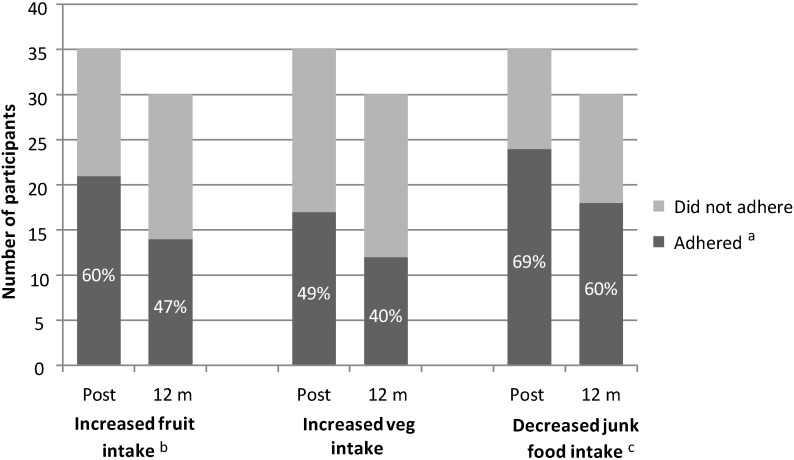
Adherence to the key CAFAP intervention messages regarding increasing intake of fruit and vegetables and decreasing intake of junk food in a group of 35 overweight adolescents. ^a^ Adherence data taken from three day food records; ^b^ An increased intake was defined as in increase in fruit or vegetable consumption by at least 0.25 servings; ^c^ A reduced intake was defined as a reduction in junk food consumption by at least 0.5 servings.

Data from the post-intervention food records showed 21 out of 35 participants who completed the eight week program adhered to the dietary intervention messages by increasing their fruit intake by at least 0.25 of a serve from pre-intervention levels. For vegetables, 17 out of 35 participants who completed the program increased their intake by at least 0.25 of a serve and 24 out of 35 participants reduced their junk food intake by at least 0.5 of a serve. The rate of adherence was reduced at 12 months post-intervention (see Figure 2).

Of the 24 participants who had complete data at both time points, 13 adhered to the fruit message at post-intervention and 10 adhered at 12 months post-intervention. Similarly, 12 of 24 participants adhered to the vegetable message at post-intervention and 10 participants adhered at 12 months post-intervention. For junk food, 18 of 24 participants adhered by reducing their junk food intake and 14 adhered at 12 months post-intervention. Adherence to the ‘reduce your junk food intake’ message had the highest proportion of adherence across the measurement period, followed by ‘increase your fruit intake’ and lastly ‘increase your vegetable intake’.

### 3.2. Eating Behaviors

The changes in self-reported eating behaviors at each time point and the monthly rate of change over each assessment period can be seen in Table 1. As expected, self-reported dietary behaviors were stable during the waitlist period (between baseline and pre-intervention). Significant improvements in frequency of breakfast consumption were reported between pre-intervention and three months post-intervention (estimated change 0.4 points, 95% CI: 0.12, 0.83). Reductions in reported fast food consumption were significantly different to pre-intervention levels at 3 months (−0.20 points, CI: −0.38, −0.02), six months (−0.24 points, CI: −0.41, −0.06) and 12 months post-intervention (−0.28 points, CI: −0.52. −0.03). Similarly, the frequency of sugar sweetened beverage consumption was significantly less than pre-intervention at six months (−0.41 points, CI: −0.71, −0.10) and 12 months post-intervention (−0.53 points, CI: −0.91, 0.15). The monthly rate of change for fast food and sugar sweetened beverage consumption did not differ from the monthly change observed during the waitlist period. Self-reported changes in fruit and vegetable consumption from the eating behavior questionnaire suggested significant increases in intake at each time point following intervention (see Table 1), although no changes were detected in reported junk food intake. The rate of change of consumption measured during the intervention period was significantly different to the waitlist period for fruit (0.17 servings per day/month, CI: 0.06, 0.28) and vegetables (0.25 servings per day/month, CI: 0.07, 0.42). Changes in the frequency of dinner consumption showed a significant monthly improvement between six and 12 months (0.04 points/month, CI: 0.01, 0.07). There were no changes detected for frequency of eating dinner as a family or eating dinner in front of the television.

### 3.3. Detailed Nutrient Intakes

The changes in nutrient intake at each time point and the monthly rate of change over each assessment period can be seen in Table 2. During the waitlist period there were no changes in energy, fat or saturated fat intake, nor any changes in the percent of energy provided by fat, saturated fat or protein. Intake of key micronutrients (zinc, calcium, iron, Vitamin C) did not change over the waitlist period. There was a reported increase in consumption of protein (69.1, SE 1.4 g/day to 74.5, SE 1.8 g/day, *p* = 0.029), and reduction in consumption of carbohydrates (202.1, SE 3.6 g/day to 188.9, SE 3.9 g/day, *p* = 0.028) and sugar (88.4, SE 3.5 g/day to 76.2, SE 3.2 g/day, *p* = 0.018) during the waitlist period. A reduction in the percent total energy provided by carbohydrates was also observed (46.6%, SE 0.6% to 44.6%, SE 0.7%, *p* = 0.046).

**Table 1:** Mean self-reported eating behavior point estimates and rates of change across intervention and follow up in a cohort of 58 overweight adolescents.

		Mean (SE) *	Period of Change	Mean Δ per Month (95% CI)	*p*-value Compared to Baseline to Pre ^d^
Frequency of breakfast	*Baseline*	3.0 (0.1)			
	*Pre*	2.9 (0.1)	*Baseline to Pre*	−0.03 (−0.13, 0.08)	ref
	*Post*	3.1 (0.1)	*Pre to Post*	0.13 (−0.04, 0.29)	0.208
	*3 months*	3.4 (0.1) ^a,b^	*Post to 3 m*	0.07 (−0.03, 0.18)	0.180
	*6 months*	3.2 (0.2)	*3 m to 6 m*	−0.05 (−0.18, 0.08)	0.793
	*12 months*	2.8 (0.2)	*6 m to 12 m*	0.07 (−0.14, −0.004) ^c^	0.475
	***Maintenance***		*Post-12 m*	−0.03 (−0.06, 0)	0.958
Frequency of fast food	*Baseline*	0.6 (0.05)			
	*Pre*	0.5 (0.1)	*Baseline to Pre*	−0.01 (−0.06, 0.04)	ref
	*Post*	0.4 (0.1)	*Pre to Post*	−0.05 (−0.15, 0.06)	0.578
	*3 months*	0.3 (0.1) ^a,b^	*Post to 3 m*	−0.03 (−0.11, 0.05)	0.639
	*6 months*	0.3 (0.1) ^a,b^	*3 m to 6 m*	−0.01 (−0.08, 0.05)	0.972
	*12 months*	0.3 (0.1) ^a,b^	*6 m to 12 m*	−0.01 (−0.05, 0.04)	0.881
	***Maintenance***			−0.01 (−0.04, 0.01)	0.907
Frequency of sweetened beverages	*Baseline*	1.5 (0.1)			
	*Pre*	1.3 (0.1)	*Baseline to Pre*	−0.04 (−0.13, 0.05)	ref
	*Post*	1.1 (0.1) ^a^	*Pre to Post*	−0.14 (−0.03, 0.03)	0.353
	*3 months*	1.1 (0.1) ^a^	*Post to 3 m*	0.01 (−0.10, 0.12)	0.521
	*6 months*	0.9 (0.1) ^a,b^	*3 m to 6 m*	−0.06 (−0.17, 0.07)	0.800
	*12 months*	0.8 (0.1) ^a,b^	*6 m to 12 m*	−0.02 (−0.09, 0.05)	0.759
	***Maintenance***		*Post to 12 m*	−0.02 (−0.05, 0.01)	0.741
Perceived daily fruit serves	*Baseline*	1.6 (0.1)			
	*Pre*	1.5 (0.1)	*Baseline to Pre*	−0.03 (−0.10, 0.04)	ref
	*Post*	1.9 (0.1) ^a,b^	*Pre to Post*	0.17 (0.06, 0.28) ^c^	0.011
	*3 months*	1.8 (0.1) ^b^	*Post to 3 m*	−0.02 (−0.10, 0.06)	0.892
	*6 months*	1.8 (0.1) ^b^	*3 m to 6 m*	−0.01 (−0.09, 0.06)	0.785
	*12 months*	1.9 (0.1) ^b^	*6 m to 12 m*	0.01 (−0.04, 0.06)	0.333
	***Maintenance***		*Post to 12 m*	0 (−0.03, 0.02)	0.476
Perceived daily vegetable serves	*Baseline*	2.5 (0.1)			
	*Pre*	2.4 (0.1)	*Baseline to Pre*	−0.03 (−0.13, 0.07)	ref
	*Post*	2.9 (0.1) ^a,b^	*Pre to Post*	0.25 (0.07, 0.42) ^c^	0.022
	*3 months*	3.0 (0.1) ^a,b^	*Post to 3 m*	0.02 (−0.11, 0.15)	0.557
	*6 months*	3.1 (0.1) ^a,b^	*3 m to 6 m*	0.05 (−0.08, 0.17)	0.351
	*12 months*	3.3 (0.2) ^a,b^	*6 m to 12 m*	0.03 (−0.05, 0.11)	0.348
	***Maintenance***		*Post to 12 m*	0.03 (−0.01, 0.08)	0.272
Perceived daily junk food serves	*Baseline*	1.6 (0.1)			
	*Pre*	1.7 (0.1)	*Baseline to Pre*	0.03 (−0.08, 0.15)	ref
	*Post*	1.5 (0.1)	*Pre to Post*	−0.07 (−0.21, 0.06)	0.331
	*3 months*	1.4 (0.1)	*Post to 3 m*	−0.03 (−0.13, 0.07)	0.437
	*6 months*	1.5 (0.1)	*3 m to 6 m*	0.07 (−0.04, 0.19)	0.617
	*12 months*	1.6 (0.2)	*6 m to 12 m*	−0.01 (−0.08, 0.06)	0.551
	***Maintenance***		*Post to 12 m*	0.01 (−0.03, 0.04)	0.667

^*^ SE is standard error; ref is the reference period; ^a^ Difference from baseline (*p* < 0.05); ^b^ difference from pre (*p* < 0.05); ^c^ significant rate of change between the two assessment points (*p* < 0.05); ^d^ This column identifies significant differences (*p* < 0.05) in the rate of change between the time period being measured and the waitlist control period (baseline to pre-intervention); Maintenance: The period between post-intervention and 12 months post-intervention; *n* = 58.

**Table 2:** Mean nutrient intake (point estimates) and rates of change across intervention and follow up in a cohort of 58 overweight adolescents.

		Mean (SE) *	Period of Change	Mean Δ per Month (95% CI)	*p*-value Compared to Baseline to Pre ^d^
Energy (kJ)	*Baseline*	6969 (46.9)			
	*Pre*	6972 (50.8)	*Baseline to Pre*	1.0 (−50.5, 52.4)	ref
	*Post*	6965 (60.0)	*Pre to Post*	−3.2 (−86.9, 80.3)	0.942
	*3 months*	6946 (58.2)	*Post to 3 m*	−6.7 (−58.4, 45.0)	0.834
	*6 months*	6987 (68.2)	*3 m to 6 m*	13.9 (−43.1, 71.0)	0.745
	*12 months*	7133 (86.0)	*6 m to 12 m*	24.2 (−10.4, 58.8)	0.473
	***Maintenance***		*Post to 12 m*	13.9 (−2.2, 30.0)	0.644
Protein (g)	*Baseline*	69.1 (1.4)			
	*Pre*	74.5 (1.8) ^a^	*Baseline to Pre*	1.8 (0.2, 3.4) ^c^	ref
	*Post*	75.3 (2.3) ^a^	*Pre to Post*	0.4 (−2.7, 3.4)	0.489
	*3 months*	76.1 (2.1) ^a^	*Post to 3 m*	0.3 (−1.7, 2.2)	0.213
	*6 months*	76.6 (2.2) ^a^	*3 m to 6 m*	0.2 (−1.7, 2.0)	0.189
	*12 months*	72.2 (2.5) ^a^	*6 m to 12 m*	−0.7 (−1.8, 0.4)	0.010
	***Maintenance***	.	*Post to 12 m*	−0.3 (−0.8, 0.3)	0.015
Fat (g)	*Baseline*	63.4 (1.3)			
	*Pre*	66.0 (1.3)	*Baseline to Pre*	0.8 (−0.5, 2.2)	ref
	*Post*	59.3 (1.6) ^b^	*Pre to Post*	−3.3 (−5.4, −1.2) ^c^	0.005
	*3 months*	62.6 (1.6)	*Post to 3 m*	1.1 (−0.3, 2.5)	0.800
	*6 months*	62.8 (1.8)	*3 m to 6 m*	0.1 (−1.4, 1.5)	0.432
	*12 months*	64.7 (1.8)	*6 m to 12 m*	0.3 (−0.5, 1.1)	0.489
	***Maintenance***		*Post to 12m*	0.5 (0.1, 0.8) ^c^	0.568
Saturated fat (g)	*Baseline*	26.9 (0.8)			
	*Pre*	27.5 (0.8)	*Baseline to Pre*	0.2 (−0.6, 0.9)	ref
	*Post*	23.6 (0.9) ^a b^	*Pre to Post*	−2.0 (−3.1, −0.8) ^c^	0.011
	*3 months*	25.4 (0.9)	*Post to 3 m*	0.6 (−0.2, 1.4)	0.416
	*6 months*	25.8 (0.8)	*3 m to 6 m*	0.1 (−0.6, 0.9)	0.939
	*12 months*	26.5 (0.8)	*6 m to 12 m*	0.1 (−2.5, 0.4)	0.854
	***Maintenance***		*Post to 12 m*	0.2 (0.1, 0.4) ^c^	0.876
Carbo-hydrate (g)	*Baseline*	202.1 (3.6) ^b^			
	*Pre*	188.9 (3.9) ^a^	*Baseline to Pre*	−4.4 (−8.3, −0.5) ^c^	ref
	*Post*	199.4 (4.8) ^a^	*Pre to Post*	5.2 (−1.3, 11.8)	0.032
	*3 months*	189.1 (4.0) ^a b^	*Post to 3 m*	−3.4 (−7.6, 0.7)	0.739
	*6 months*	190.0 (5.4) ^a b^	*3 m to 6 m*	0.3 (−3.8, 4.4)	0.110
	*12 months*	196.1 (4.7) ^a b^	*6 m to 12 m*	1.0 (−1.1, 3.2)	0.018
	***Maintenance***		*Post to 12 m*	−0.3 (−1.3, 0.8)	0.048
Sugar (g)	*Baseline*	88.4 (3.5)			
	*Pre*	76.2 (3.2) ^a^	*Baseline to Pre*	−4.1 (−7.5, −0.7) ^c^	ref
	*Post*	83.6 (5.5)	*Pre to Post*	3.7 (−2.9, 10.4)	0.066
	*3 months*	81.3 (4.1)	*Post to 3 m*	−0.8 (−5.3, 3.8)	0.250
	*6 months*	77.4 (4.9)	*3 m to 6 m*	−1.3 (−5.3, 2.7)	0.300
	*12 months*	82.7 (4.6)	*6 m to 12 m*	0.9 (−1.2, 3.0)	0.014
	***Maintenance***		*Post to 12 m*	−0.1 (−1.1, 1.0)	0.027
Fiber (g)	*Baseline*	16.1 (0.4)			
	*Pre*	15.2 (0.5)	*Baseline to Pre*	−0.3 (−0.7, 0.1)	ref
	*Post*	16.8 (0.5) ^b^	*Pre to Post*	0.8 (0.1, 1.4) ^c^	0.017
	*3 months*	16.8 (0.6) ^b^	*Post to 3 m*	0 (−0.5, 0.5)	0.326
	*6 months*	18.4 (0.8) ^a b^	*3 m to 6 m*	0.5 (−0.03, 1.1)	0.016
	*12 months*	17.1 (0.6) ^b^	*6 m to 12 m*	−0.2 (−0.5, 0.1)	0.692
	***Maintenance***		*Post to 12 m*	0.03 (−0.1, 0.2)	0.123
kJ from protein (%)	*Baseline*	17.5 (0.3)			
	*Pre*	18.4 (0.4)	*Baseline to Pre*	0.3 (−0.04, 0.7)	ref
	*Post*	19.1 (0.5) ^a^	*Pre to Post*	0.4 (−0.3, 1.1)	0.869
	*3 months*	19.2 (0.5) ^a^	*Post to 3 m*	0.02 (−0.4, 0.5)	0.327
	*6 months*	19.4 (0.5) ^a^	*3 m to 6 m*	0.05 (−0.4, 0.5)	0.364
	*12 months*	18.2 (0.6)	*6 m to 12 m*	−0.2 (−0.5, 0.1)	**0.022**
	***Maintenance***		*Post to 12 m*	−0.1 (−0.2, 0.05)	**0.037**
kJ from fat (%)	*Baseline*	33.1 (0.6)			
	*Pre*	34.8 (0.6)	*Baseline to Pre*	0.5 (−0.04, 1.1)	ref
	*Post*	31.2 (0.8) ^b^	*Pre to Post*	−1.7 (−2.7, −0.8) ^c^	**0.001**
	*3 months*	33.9 (0.8)	*Post to 3 m*	0.9 (0.1, 1.6) ^c^	0.471
	*6 months*	33.5 (1.0)	*3 m to 6 m*	−0.1 (−1.0, 0.7)	0.212
	*12 months*	34.1 (1.0)	*6 m to 12 m*	0.1 (−0.4, 0.5)	0.224
	***Maintenance***		*Post to 12 m*	0.2 (0.05, 0.4) ^c^	0.343
kJ from sat fat (%)	*Baseline*	14.0 (0.4)			
	*Pre*	14.4 (0.3)	*Baseline to Pre*	0.1 (−0.2, 0.4)	ref
	*Post*	12.3 (0.4) ^a b^	*Pre to Post*	−1.1 (−1.6, −0.5) ^c^	**0.002**
	*3 months*	13.8 (0.5)	*Post to 3 m*	0.5 (0.1, 1.0) ^c^	0.146
	*6 months*	13.8 (0.4)	*3 m to 6 m*	0.02 (−0.5, 0.5)	0.706
	*12 months*	14.0 (0.4)	*6 m to 12 m*	0.03 (−0.2, 0.2)	0.611
	***Maintenance***		*Post to 12 m*	0.1 (0.1, 0.2) ^c^	0.902
kJ from Carbo-hydrate (%)	*Baseline*	46.6 (0.6)			
	*Pre*	44.6 (0.7) ^a^	*Baseline to Pre*	−0.7 (−1.3, −0.01) ^c^	ref
	*Post*	47.0 (1.0)	*Pre to Post*	1.2 (−0.1, 2.5)	**0.032**
	*3 months*	44.0 (0.9) ^a^	*Post to 3 m*	−1.0 (−2.0, −0.1) ^c^	0.547
	*6 months*	44.3 (1.0)	*3 m to 6 m*	0.1 (−0.8, 1.0)	0.164
	*12 months*	44.7 (1.2)	*6 m to 12 m*	0.1 (−0.5, 0.6)	0.093
	***Maintenance***		*Post to 12 m*	−0.2 (−0.5, 0.1)	0.181
Calcium (mg)	*Baseline*	601.8 (22.8)			
	*Pre*	598.9 (25.0)	*Baseline to Pre*	−1.0 (−24.9, 22.9)	ref
	*Post*	606.2 (25.5)	*Pre to Post*	3.6 (−33.5, 40.7)	0.864
	*3 months*	663.6 (30.5)	*Post to 3 m*	19.1 (−6.5, 44.7)	0.255
	*6 months*	646.6 (37.9)	*3 m to 6 m*	−5.7 (−35.2, 23.9)	0.811
	*12 months*	663.9 (34.0)	*6 m to 12 m*	2.9 (−13.8, 19.5)	0.784
	***Maintenance***		*Post to 12 m*	4.8 (−1.9, 11.5)	0.635

^*^ SE is standard error; ref is the reference period; ^a^ Difference from baseline (*p* < 0.05); ^b^ difference from pre (*p* < 0.05); ^c^ significant rate of change between the two assessment points (*p* < 0.05); ^d^ This column identifies significant differences (*p* < 0.05) in the rate of change between the time period being measured and the waitlist control period (baseline to pre-intervention); Maintenance: The period between post-intervention and 12 months post-intervention; *n* = 58.

Following the eight-week intervention, there was a significant reduction in point estimates of fat (66.0, SE 1.3 g/day to 59.3, SE 1.6 g/day, *p* = 0.002) and saturated fat consumption (27.5, SE 0.8 g/day to 23.6, SE 0.9 g/day, *p* = 0.001). The rate of change of fat (−3.3 g per day per month, 95%CI: −5.4, −1.2, *p* = 0.005) and saturated fat consumption (−2.0 g per day per month, 95%CI: −3.1, −0.8, *p* = 0.011) was significantly improved from the rate of change during the waitlist period. A reduction in the percentage total energy provided by fat (34.8%, SE 0.6% to 31.2%, SE 0.8%, *p* ≤ 0.001) and saturated fat (14.4%, SE 0.3% to 12.3%, SE 0.4%, *p* ≤ 0.001) was also observed, along with a significantly improved monthly rate of change during the intervention period compared to the waitlist period (see Table 2). There were no changes in energy, protein or sugar intake during intervention. Point estimates of fiber were significantly increased (15.2, SE 0.5 g/day to 16.8, SE 0.6 g/day, *p* = 0.016) and the monthly rate of change of fiber (0.8 g per day per month, 95%CI: 0.1, 1.4, *p* = 0.017) was significantly more than that observed during the waitlist period.

During the 12-month maintenance period nutrient intakes appeared to regress towards baseline levels (see Table 2). Point estimates of fat and saturated fat between three and 12 months post-intervention were no longer different to pre-intervention levels. The percent energy provided by macronutrients during the maintenance period was not different to pre-intervention distributions. There was a significant increase in the percent of energy provided by fat between post-intervention and three months post-intervention (31.2, SE 0.8% to 33.9 SE 0.8%, *p* = 0.022) with a significant increase in the monthly rate of change (*p* = 0.002). Total carbohydrate intake did remain lower than pre-intervention levels throughout the 12 month maintenance period and fiber intake remained significantly higher than pre-intervention levels (see Table 2). Energy, protein and sugar intake did not change during the maintenance period, nor did intakes of calcium, zinc or vitamin C.

## 4. Discussion

This study provides unique data on adherence to the dietary component of a multi-component intervention in obese adolescents and is one of the few adolescent intervention studies to consider eating behavior and dietary intake changes in the 12 month period following intervention. Further, this study used three day food records to provide detailed dietary change data for overweight adolescents. The main findings were that a large proportion of participants adhered to the key components of the dietary intervention, with modest dietary changes seen following intervention and lessening over time.

### 4.1. Adherence

More than half of the 35 participants who completed this study adhered to the key intervention messages about eating more fruit and vegetables and eating less junk food, but the percentage who adhered reduced over time. There is a lack of prior studies in overweight adolescents that incorporated any measures of adherence to dietary interventions, preventing comparisons to these results [18]. This gap in adherence measurement has begun to be addressed in this study by using data from detailed food records to measure adherence to the CAFAP dietary intervention messages. The methods used have been developed to suit the study given the lack of previous reporting of adherence in overweight adolescent interventions and the absence of guidelines regarding the best way to measure or report dietary adherence in intervention studies [41].The interpretation of the adherence levels in this study is further limited by the lack of evidence regarding what constitutes satisfactory levels of adherence. Previous studies have identified a range of 80%–120% of recommended nutrient intakes as an indicator of adherence [42]. This method was not appropriate in the current study, given that adolescents were encouraged to improve their intake rather than achieve ideal but perhaps unrealistic diet goals. Other methods from previous research measuring dichotomous variables were not applicable due to the multi-factorial nature of diet. Thus, the adherence reported in this paper relates to the proportion of participants who have adhered to the different dietary change messages. Future interventions can compare their findings to these levels of adherence and work towards a clear consensus for acceptable levels of adherence to dietary interventions based on observed changes in health status.

Behavior change as a result of the CAFAP dietary intervention was assessed by adherence to the CAFAP dietary intervention messages. It was hypothesized that targeting and improving key theoretical constructs, such as motivation and parent support, would lead to dietary behavior change [43] based on self-determination and goal setting theories [23]. However, exploratory post hoc analysis did not support this relationship. When autonomous motivation for healthy eating and perceived parental support for healthy eating were compared between those who adhered to key dietary messages and those did not adhere, there were no significant differences between those groups at either post-intervention or 12-months post-intervention. Due to the limited sample size, it was not possible to complete a full mediation analysis; however, future research should include mediation analyses of the theoretical constructs to help explain the mechanisms for successful behavior change.

### 4.2. Eating Behaviors

There were some changes in self-reported eating behaviors during the maintenance period including increased consumption of breakfast and reduced consumption of fast food and SSBs. CAFAP participants reported changes in line with prior studies of overweight adolescents, in both fast food intake [44] and SSB intake [45]. Whilst these behavior changes remained significant for CAFAP participants during the maintenance period, comparisons of sustained change are limited as no other studies have reported general eating behavior changes for at least 12 months following intervention. Thus, these findings add much-needed data to the limited evidence base around overweight adolescent eating behaviors, and provide an example of how these may change following intervention.

### 4.3. Nutrient Intakes

The modest dietary changes observed in this study, reductions in fat and saturated fat and an increase in fiber; reflect the current evidence from obesity interventions. CAFAP participants reported no changes in total energy intake compared to the waitlist control period, reflecting other recent trials where energy intake did not differ from the control comparison [14,45,46,47,48]. A recent trial demonstrated significant reductions in adolescent energy, fat and saturated fat consumption immediately post-intervention, but did not include any control group or waitlist comparison [16]. A significant reduction in adolescent total fat intake has also been previously reported, but based on a brief questionnaire not yet validated in adolescents [12]. Neither of these studies adjusted for underreporting and neither showed a reduction in percentage of total energy provided by fat as CAFAP did, which is thought to be a more reliable measure than absolute fat intake [49]. In a study of Latino adolescents, participants reported significant reductions in total sugar intake in one of two intervention groups immediately post-intervention [19], although these results may not be generalizable given both groups had received the same nutrition component of the intervention. There were no changes in reported sugar consumption following the CAFAP intervention. 

Further, findings showed micronutrient levels did not change throughout the study. This suggests that CAFAP did not have a deleterious effect on nutritional intake throughout the study period. Similar results have been found in other studies with some measure of nutrient intake, with no reductions in key nutrients following intervention [16,45,50]. Calcium intake, important for growth and development, was consistently low throughout the current study (~600–650 mg), which might suggest an important potential target for future dietary interventions for adolescents.

The gradual pattern for macronutrient intake levels to regress towards baseline intake levels over the 12 month maintenance period may reflect the waning adherence to CAFAP nutrition intervention messages. This pattern was also reflected in the regression of physical activity changes [39]. The loss of changes occurred alongside the tapering maintenance support provided to adolescents, aspects of which were not well-received in the initial three months post-intervention [51]. This might suggest that future programs would benefit from more intensive support over 12 months, possibly using a mode of contact other than text messaging. Despite a loss of positive changes in macronutrient intakes during the maintenance period, these levels did not worsen from baseline levels. Future trials should monitor long-term dietary changes to understand how well dietary change is sustained, and plan maintenance support programs accordingly.

### 4.4. Dietary Assessment Methods

In this study, adolescents rated their own intake of fruits and vegetables differently using the short food behavior questionnaire compared to how they recorded their intake using a three day food record. Despite the reducing levels of adherence and modest changes in diet, as shown by the food records, the estimates of fruit and vegetable intake from the eating behavior questionnaire remained significantly increased following intervention for the entire 12 month maintenance period. For example, the food records showed no change in vegetable intake following intervention but the short questionnaire showed an increase of vegetable intake during the maintenance period of up to 0.9 serves at 12 months post-intervention. Thus, it seems that the food behavior questionnaire may have overestimated the effect of the intervention on intakes as compared to the food records, particularly for vegetables. This discrepancy may be due to a desire to report socially acceptable intakes in line with the CAFAP key messages [52], particularly given that all changes occurred after the intervention had been delivered. The wording of the questions directly reflected the nutrition intervention messages, so adolescents may have felt obliged to show they had adhered. Alternatively the participants may have truly believed that they were eating more healthfully. The differences in self-reported behaviors from questionnaire and self-reported intake from food records highlight the inherent difficulties in obtaining consistent and accurate nutrition data in this population.

### 4.5. Strengths and Limitations

The strengths of the study include the use of multiple dietary assessment measures, detailed description of obese adolescent dietary change following intervention, detailed maintenance dietary data for a further 12 months and adjustments for the impact of underreporting. The use of objective accelerometry data in the estimation of total energy expenditure provides added confidence in the adjustment for underreporting. Three day food records were used in this study to provide detailed information about consumption patterns, including timing of meals [53], without being limited by extensive reliance on memory and time available for physical assessment. Although three day food records have known limitations with potential underreporting, in this study we were able to use underreporting as a covariate to control for the effect of underreporting on the dietary outcomes, giving greater confidence in the results. Aside from the limitations associated with any assessment of self-reported diet, other limitations included a relatively small sample size. Due to recruitment difficulties and issues with retention in the study, 69 participants were included in the sample size at baseline, which is less than initially planned. There were further issues with drop outs during the study, although the attrition rate of 51% is in line with other recent pediatric weight management literature [40]. The proportionately low numbers of male participants in this study reduces the generalizability of these findings. Additional studies are needed to replicate these findings in diverse populations.

## 5. Conclusions 

This is one of the first studies to report overweight and obese adolescent adherence to the dietary component of a multi-component lifestyle intervention. Findings showed that adherence rates were highest for CAFAP messages about reducing junk food. Overweight and obese adolescents who participated in CAFAP reported modest improvements in some key eating behaviors and nutrient intakes, although this differed between methods of dietary assessment. The brief eating behavior questionnaire gave a potentially more positive impression about the adolescent dietary response to intervention, and so this data should be viewed with caution. Future studies with overweight and obese adolescents should report adherence to dietary interventions and use standardized and practical methods for assessing and controlling for underreporting. These data provide evidence to support the call for more comprehensive and long-term reporting of dietary intake in obese adolescent interventions to better understand dietary changes in this group and, thus, guide the design of effective interventions.
